# Nestin-positive mesenchymal stem cells favour the astroglial lineage in neural progenitors and stem cells by releasing active BMP4

**DOI:** 10.1186/1471-2202-5-33

**Published:** 2004-09-15

**Authors:** Sabine Wislet-Gendebien, Françoise Bruyère, Grégory Hans, Pierre Leprince, Gustave Moonen, Bernard Rogister

**Affiliations:** 1 Centre for Cellular and Molecular Neurobiology, University of Liège, Liège, Belgium; 2 Department of Neurology, C.H.U. of Liège, Sart Tilman, Belgium

## Abstract

**Background:**

Spontaneous repair is limited after CNS injury or degeneration because neurogenesis and axonal regrowth rarely occur in the adult brain. As a result, cell transplantation has raised much interest as potential treatment for patients with CNS lesions. Several types of cells have been considered as candidates for such cell transplantation and replacement therapies. Foetal brain tissue has already been shown to have significant effects in patients with Parkinson's disease. Clinical use of the foetal brain tissue is, however, limited by ethical and technical problems as it requires high numbers of grafted foetal cells and immunosuppression. Alternatively, several reports suggested that mesenchymal stem cells, isolated from adult bone marrow, are multipotent cells and could be used in autograft approach for replacement therapies.

**Results:**

In this study, we addressed the question of the possible influence of mesenchymal stem cells on neural stem cell fate. We have previously reported that adult rat mesenchymal stem cells are able to express nestin in defined culture conditions (in the absence of serum and after 25 cell population doublings) and we report here that nestin-positive (but not nestin-negative) mesenchymal stem cells are able to favour the astroglial lineage in neural progenitors and stem cells cultivated from embryonic striatum. The increase of the number of GFAP-positive cells is associated with a significant decrease of the number of Tuj1- and O4-positive cells. Using quantitative RT-PCR, we demonstrate that mesenchymal stem cells express LIF, CNTF, BMP2 and BMP4 mRNAs, four cytokines known to play a role in astroglial fate decision. In this model, BMP4 is responsible for the astroglial stimulation and oligodendroglial inhibition, as 1) this cytokine is present in a biologically-active form only in nestin-positive mesenchymal stem cells conditioned medium and 2) anti-BMP4 antibodies inhibit the nestin-positive mesenchymal stem cells conditioned medium inducing effect on astrogliogenesis.

**Conclusions:**

When thinking carefully about mesenchymal stem cells as candidates for cellular therapy in neurological diseases, their effects on resident neural cell fate have to be considered.

## Background

During development of the central nervous system (CNS), all types of neuronal and macroglial cells derive from neuroepithelial neural stem cells (NSCs) [1,2]. NSCs self-renew and continue to function as source of new cells in adults [3,4]. The fate determination of neural stem cells is regulated by cell-intrinsic programs as well as extrinsic cues from the surrounding environment [5]. In the adult, the molecular mechanisms that regulate the production of new neurons in the dentate gyrus or in the subventricular zone are still unknown, although extrinsic factors expressed for example by astrocytes could play a role [6].

BMPs (Bone Morphogenetic Proteins) are secreted members of the TGF-β superfamily. Together with their receptors, they are abundantly expressed in the brain both during embryogenesis and in the adult [7-9]. More specifically, an expression of BMP4 was demonstrated in different cell types like ectodermal cells [10], radial glia [11], haematopoietic cells [12], chondrocytes [13] and stromal cells [14]. As for other members of the TGF-β superfamily, BMPs signal transduction is triggered by binding to type 1 and type 2 serine-threonine kinase receptors, inducing their dimerization. In this manner, BMP2 and BMP4 signalling specifically leads to the formation of BMPR-1A/BMPR-II and BMPR-1B/BMPR-II heterodimers [15]. BMPs have been shown to play a role in patterning and cellular fate determination in many tissues [16]. For examples, during development, BMPs are involved in the induction of the neuroectoderm, the patterning of the dorsal roof of neural tube, the development of neural crests and of the peripheral nervous system [10,17-20]. In postnatal animals, they promote the differentiation of neuronal precursors in the spinal cord [21] and in the cortex [22,23]. Finally, they facilitate an astroglial lineage commitment of forebrain subventricular zone progenitor cells [24].

Cellular therapies using stem cells are promising approaches for the treatment of several chronic or acute neurological diseases such as Parkinson's [25] or Huntington's diseases [26] or spinal cord injuries [27]. One main problem relates to the origin and the nature of the cells to be used for such procedures. Foetal brain tissue has already been shown to have significant effects in patients with Parkinson's disease. Clinical use of the foetal brain tissue is, however, limited by ethical and technical problems as it requires high numbers of grafted foetal cells and a possible immunosuppression. Alternatively, somatic stem cells derived from adult tissues seem to be better candidates for cell replacement therapy [28,29]. These observations raise hopes in cell replacement strategies based on an autograft approach. However, the exact mechanism by which mesenchymal stem cells (MSCs) adopt a neural fate is not completely understood as recent *in vivo *studies demonstrated that MSCs fuse with host neuronal cells [30,31]. These observations impose a better knowledge of the mechanisms underlying the phenotypic plasticity of somatic stem cells and the characterization of their daughter differentiated cells is a prerequisite before considering their use in the treatment of human patients.

Recently, we demonstrated that two phenotypes of MSCs could be obtained in culture: nestin-positive MSCs (npMSCs) which are able to integrate some extrinsic signals when co-cultured with neurons leading to a differentiation into astrocyte-like cells and nestin-negative MSCs (nnMSCs) which are unable to adopt a neural phenotype but remain able to differentiate into adipocytes, chondrocytes or osteocytes [32]. When considering the use of MSCs as a source of material for cell replacement protocols in neurological diseases, one has also to be concerned by a possible effect of these MSCs on the host nervous tissue and more particularly on immature resident neural cells. In several models of neurological diseases, grafted MSCs were shown to favour host CNS regeneration rather than to express themselves a neural phenotype [33,34]. This positive effect of MSC grafts could result from the release of factors acting on resident immature cells in the adult brain. Recently, it has been demonstrated that following ischemia in the adult striatum, intra-ventricular EGF injections are able to stimulate neurogenesis from resident neural stem cells, although EGF is devoid of any effect in non-ischemic striatum [35]. These observations emphase the importance of the lesion priming in order to respond to extrinsic factors or cytokines. In this paper, we address the question of the influence of MSCs on neural stem cells *in vitro *and demonstrate that MSCs favour astroglial lineage. We observe that npMSCs are able to stimulate astroglial fate in striatal progenitor cultures and to repress neuronal and oligodendroglial fate through the release of diffusible factor(s). Using BrdU incorporation, we demonstrate that this npMSC conditioned medium has no effect on the astrocytic or oligodendrocytic progenitor proliferation. Propidium iodide incorporations suggest that the npMSCs conditioned medium protect GFAP-positive cells from cell death in comparison to the effect of nnMSCs conditioned medium or to the control condition. Meanwhile, no increase of cell death is observed in neuronal and oligodendroglial populations. We then demonstrated that BMP4 is present in a biologically-active form in the npMSCs but not in nnMSCs conditioned medium and is responsible for both the increase of astroglial numbers and the inhibition of oligodendrocyte differentiation in striatal NSC cultures.

## Results

### Nestin-positive MSCs increase astrocytes number in differentiating neural stem cell cultures

**Figure 1:**
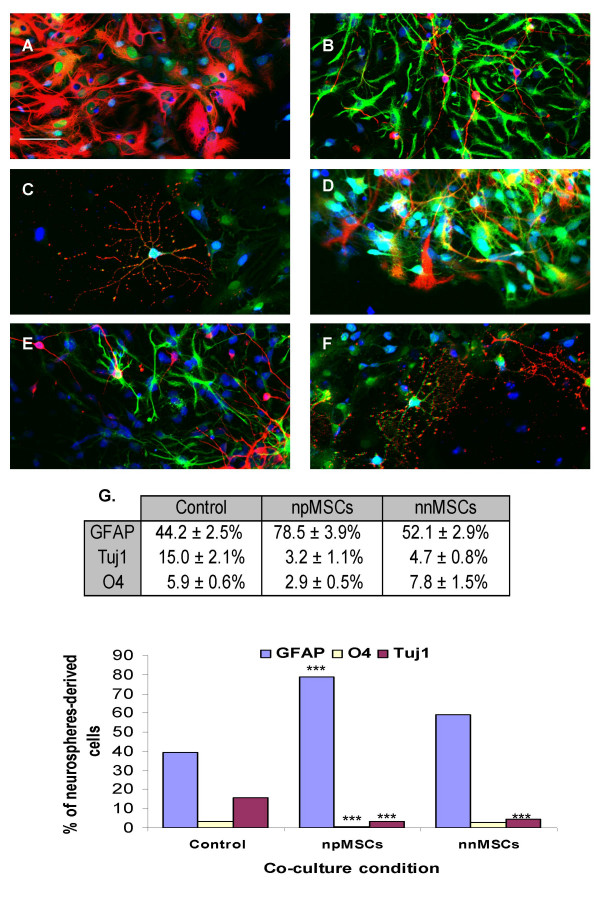
**Nestin-positive MSCs increase astrocytes number in differentiating neural stem cell cultures. **Neurosphere-derived GFP-expressing cells were co-cultivated with npMSCs or nnMSCs on polyornithine-coated dishes. After 5 days, the cells were fixed and immunostained with antibodies raised against GFAP (A, D, red), Tuj1 (B, E, red) and O4 (C, F, red). Nuclei were counterstained with EtD1 (blue). Co-cultures with npMSCs are illustrated in A (n = 12, ***Student T-test, P < 0.001), B (n = 12, ***Student T test, P < 0.0001) and C (n = 8, ***Student T test, P < 0.0001). Co-cultures with nnMSCs are illustrated in D (n = 8, Student T test, P > 0.05), E (n = 8, ***Student T test, P < 0.001) and F (n = 8, Student T test, P > 0.05). Stained cells counting results are shown in G and include the control condition of neurosphere-derived cells without co-culture. Scale bars = 20 μm in A-F.

Neurosphere-derived cells from GFP-positive E16 green mouse striata include neural stem cells and progenitors which are still proliferating but already committed to a given cell fate. Upon transfer on adherent surfaces (poly-ornithine coated dishes), these cells spread and spontaneously differentiate as follows after 5 days of culture: 44.2 ± 2.5% GFAP-positive cells (astroglial cells), 15 ± 2.1% Tuj1-positive cells (neuronal cells) and 5.86 ± 0.6% 04-positive cells (oligodendroglial cells). When co-cultivated with nestin-positive MSCs (npMSCs), phenotypic allocation of neurosphere-derived cells (identified as GFP-positive cells) become strikingly different: GFAP labelling is increased to 78.5 ± 3.9% (Fig. 1A) (n = 12, ***Student T-test, P < 0.001), while Tuj-1 and O4 are decreased to respectively 3.2 ± 1.1% (Fig. 1B) (n = 12, ***Student T test, P < 0.0001) and 2.9 ± 0.5% (Fig. 1C) (n = 8, ***Student T test, P < 0.0001). On the other hand, if neurospheres are co-cultured with nestin-negative MSCs (nnMSCs), the percentage of labelled cells were 52.1 ± 2.9%, 4.7 ± 0.8% and 7.8 ± 1.5 for respectively GFAP, Tuj-1 and O4. These numbers are not significantly different from the numbers obtained in control cultures for GFAP- and O4-positive cells (n = 8, Student T test, P > 0.05), while significantly lower for Tuj-1-postive cells (n = 8, ***Student T test, P < 0.001, Fig. 1G).

### MSCs effect on neurospheres-derived astrocytes number is due to a soluble factor

**Figure 2:**
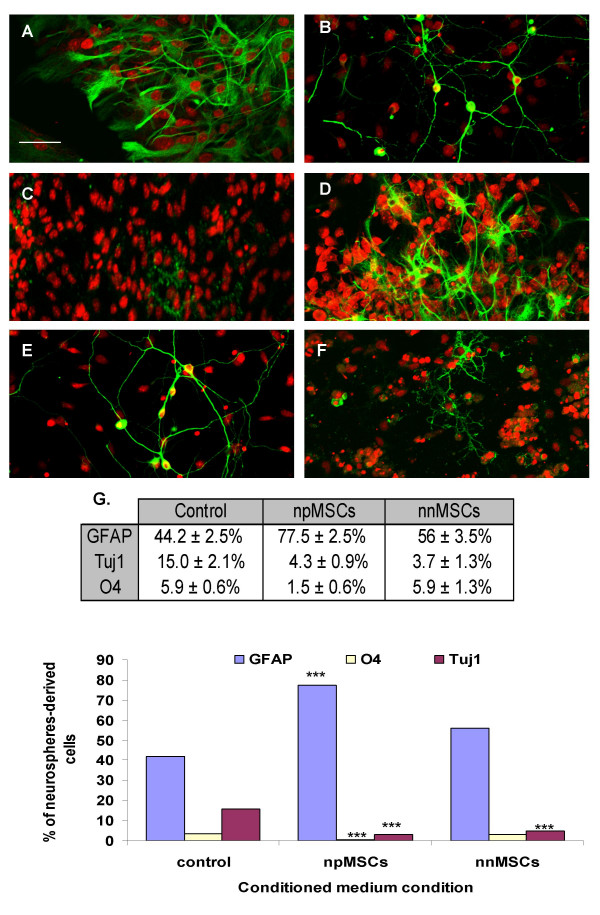
**Humoral effect of nestin-positive MSCs. **Neurosphere-derived GFP-expressing cells were cultivated with npMSC or nnMSC conditioned media, on polyornithine-coated dishes. After 5 days, the cells were fixed and immunostained with antibodies raised against GFAP (A, D, green), Tuj1 (B, E, green) and O4 (C, F, green). Nuclei were counterstained with EtD1 (red). Cultures with npMSC conditioned medium are illustrated in A, B and C. As observed in co-culture conditions, there is a significant increase of the number of GFAP-positive cells and significant decreases of the number of Tuj1-positive cells and O4-positive cells (n = 8, ***Student T test, P < 0.0001). Cultures with nnMSC conditioned medium are illustrated in D, E and F (n = 7, ***Student T test, P < 0.0001). Stained cells counting results are shown in G and include the control condition of neurosphere-derived cells without conditioned medium. No significant difference is observed between neurosphere differentiated in co-culture and in conditioned medium. (Student T test, P > 0.05). Scale bars = 20 μm in A-F.

To further characterize the mechanism (soluble factor versus membrane-bound factor) responsible for such an increase in astrocytes number in presence of npMSCs, we tried to induce the differentiation of neurospheres with npMSC conditioned medium. The following data were obtained: 77.5 ± 2.5% of the neurosphere-derived cells became GFAP-positive, 4.3 ± 0.9% were Tuj1-positive and 1.5 ± 0.6% were 04-positive (n = 8, Fig. 2A,2B,2C). Conversely, in nnMSCs conditioned medium, differentiated phenotypes were distributed as follows: 56 ± 3.5%, 3.7 ± 1.3% and 5.9 ± 1.3% (n = 7, Fig. 2G) and were similar to results obtained in corresponding co-cultures. Moreover, we confirmed those results by absolute count: 1) in npMSCs conditioned medium 392/504 cells were GFAP-positive, 9/504 cells were O4-positive and 32/504 cells were Tuj1-positive ; 2) in nnMSCs conditioned medium 251/515 cells were GFAP-positive, 29/515 were O4-positive and 23/515 were Tuj1-positive; 3) in the control condition (DEM/F12 + B27), 211/531 cells were GFAP-positive, 27/531 cells were O4-positive and 167/531 cells were Tuj1-positive. Hence, regarding the phenotypic allocation, no significant difference is observed between neurosphere differentiated in co-culture and in conditioned medium. (Student T test, P > 0.05). This clearly suggest that npMSCs-derived soluble factor(s) is (are) responsible for an increase in the astrocytes number and a decrease in the neurons and oligodendrocytes numbers.

### Nestin-positive MSC conditioned medium act on the GFAP-positve cell death

**Figure 3:**
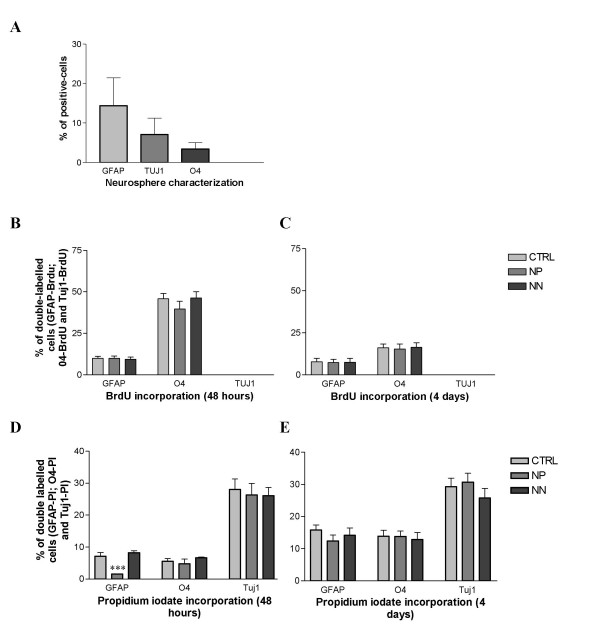
**Nestin-positive MSC conditioned medium act on the GFAP-positive cell death but not on cell proliferation. **(A) Characterization of committed cell types in proliferating neurospheres in presence of EGF. We observed that 14.4 ± 7.1%, 7.1± 4.1% and 3.4 ± 1.6% of cells were respectively GFAP-, Tuj1- and O4-positive (these data were obtained by absolute counts on 1576 cells). (B-C) The proliferative capacity of the differentiating neurosphere-derived cells placed in npMSC conditioned medium, nnMSC conditioned medium and in control medium (DEM/F12 + B27) was compared. BrdU incorporation was performed after 48 hours and 4 days in differentiating conditions. Double labelling GFAP-, Tuj1- and O4 with BrdU were performed. The BrdU incorporation by differentiating neurosphere-derived cells placed in the various conditions did not shown significant differences (Statistical test ANOVA, P > 0.05). (D-E) The cell death quantified by propidium iodide incorporation and counting was analysed after 48 hours and 4 days in differentiating conditions and in GFAP-, Tuj1- and O4-positive cells. After 48 hours, a significant decrease of the number of GFAP-positive cells which have incorporated the propidium iodide is observed (***Student T test, p < 0.0001). However, no significant increase of cell death was observed in O4- and Tuj1-positive cell population (Student T test, p > 0.05). After 4 days of differentiation, no difference in cell death is observed whatever the cell type or the culture condition.

As mentioned above, neurosphere derived-cells include neural stem cells and progenitors which are still proliferating but already committed to a given cell fate. We characterised the proportion of each progenitor cell-type present in our neurospheres. After dissociation, cells were allowed to adhere for one hour in coated dish and then fixed, labelled and counted. In those conditions we observed 14.4 ± 7.1% of GFAP-positive cells, 7.1± 4.1% of Tuj1-positive cells and 3.4 ± 1.6% of O4-positive cells (these data were obtained by absolute counts on 1576 cells, Fig. 3A).

In order to define if npMSCs conditioned medium has an instructive or/and a selective effect on neural cells, we analysed the proliferative capacity and the cell death in the differentiating neurospheres. The BrdU incorporation in differentiating neurosphere-derived cells cultivated in npMSCs- or in nnMSCs-conditioned medium or in control (non-conditioned) medium (DEM/F12 + B27) did not show any significant differences (Statistical test ANOVA, P > 0.05, Fig. 3B,3C). These data rule out a proliferative effect on already committed astrocyte progenitors or an inhibition of cell proliferation in oligodendroglial and neuronal progenitors. Furthermore, using propidium iodide incorporations and counting, we quantified the cell death in GFAP-, Tuj1- and O4-positive cell population during the differentiation culture in the three conditioned media (by npMSCs, by nnMSCs and non-conditioned). After 48 hours, we observed a significant decrease of the number of GFAP-positive cells which have incorporated the propidium iodide in npMSCs-conditioned medium (***Student T test, p < 0.0001) in comparison to the two other culture condition. This result suggests that npMSC conditioned medium partially inhibit the cell death in the GFAP-positive cell population. Nevertheless, no significant increase of cell death is observed in O4- and Tuj1-positive cell population (Student T test, p > 0.05). When propidum iodide incorporations are performed after four days of differentiation without renewing the conditioned media, no significant differences in the cell death could be observed in the three lineages, whatever the culture condition (Fig. 3D,3E).

### BMP4 is present in nestin-positive conditioned medium and is responsible for the increase of GFAP-positive cells in differentiating NSCs

**Figure 4:**
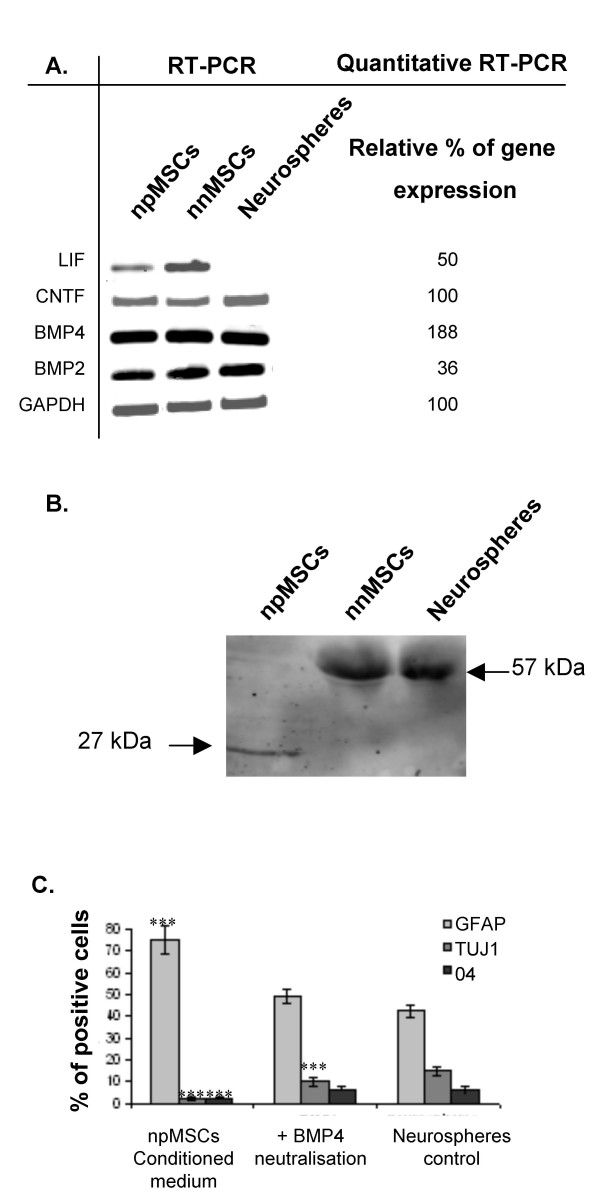
**Release of an active form of BMP4 by nestin-positive MSCs. **(A)RT-PCR and quantitative RT-PCR were performed on npMSCs and nnMSCs. The results of quantitative RT-PCR are expressed as percent of gene expression in npMSCs compared to nnMSCs (arbitrarily as 100%) after normalisation with the GAPDH house-keeping gene expression. (B) Western blotting analysis of conditioned media by npMSCs, nnMSCs and NSC. The mature and biologically-active form of BMP4 (27 kDa) is only observed in medium conditioned by npMSCs and the 57 kDa immature and non biologically-active form can be seen in nnMSC and in neural stem cells conditioned medium. This high MW form is not present in npMSC conditioned medium, indicating a complete cleavage and activation of BMP4. (C) Effect of anti-BMP4 antibodies in differentiating NSCs cultivated in DEM/F12 or npMSCs conditioned medium. Anti-BMP4 antibodies inhibit the npMSCs effect on astroglial and oligodendroglial cell numbers but not the effect on neurons number (47.13 ± 1.53% of GFAP-positive cells, 9.98 ± 0.92% of Tuj1-positive cells and 5.67 ± 0.45% of O4-positive cells with anti-BMP4 compared to respectively 77.5 ± 2.5%, 4.30 ± 0.95% and 1.47± 0.60% in npMSC conditioned-medium). Tuj1-positive cells remains significantly lower (***student T test, p < 0.001, n = 5).

Previous reports have shown that several secreted growth factors, including BMP2, BMP4, LIF and CNTF stimulate the differentiation of cultured neural precursors into astrocytes [36-41]. The expression of those cytokines was compared in nnMSCs and in npMSCs using quantitative RT-PCR. Compared to nnMSCs, we found that npMSCs slightly increased the expression level of BMP4 mRNA (190 ± 26%) while the BMP2 (36 ± 7%) and LIF (50 ± 10%) expression is slightly decreased (Fig. 4A). No significant difference was observed concerning the CNTF mRNA expression. Western blotting analyses of npMSCs, nnMSCs and neurosphere-derived cells conditioned media showed that only npMSCs release in their culture medium the mature and biologically-active form of BMP4 (27 kDa), although nnMSCs and neurosphere-derived cells expressed the biologically-inactive precursor form of BMP4 (57 kDa) (Fig. 4B). Finally, we observed that neurospheres-derived cells cultivated in npMSCs conditioned medium supplemented with an anti-BMP4 blocking antibodies, differentiate into 47.1 ± 1.5% GFAP-positive cells, 9.9 ± 0.9% Tuj1-positive cells and 5.7 ± 0.5% O4-positive cells (Fig. 4C). Statistical analyses did not show significant differences in GFAP- or O4-positive cells compared to control culture conditions (student T test, p > 0.05, n = 5). However, the number of Tuj1-positive cells remains significantly lower (***student T test, p < 0.001, n = 5). We thus conclude that the major glial effects (increase of astrocytes and decrease of oligodendrocytes) of the npMSCs on the neural progenitor differentiation is due to the release of biologically-active BMP4. The inhibition of neuronal differentiation should be attributed to another yet uncharacterized soluble factor(s).

## Discussion

During the last few years, a number of studies have addressed the phenotypic plasticity of MSCs. Most of them were performed *in vivo *and demonstrated that environmental factors play important roles in determining the ability of grafted MSCs to adopt a neural-like phenotype. Grafting of a subset of MSCs into the lateral ventricle of neonatal mice resulted in their migration within the forebrain and cerebellum, and their differentiation into astrocytes [42]. When MSCs were grafted into adult rat cerebellum after an ischemic lesion, they differentiated into cells expressing neuronal markers [43]. Nakano et al. [44] demonstrated that murine bone marrow cells differentiated into three distinct glial phenotypes (oligodendrocytes, astrocytes and microglia) when they were directly injected into the striatum of previously irradiated mice. Similarly, systemic injection of MSCs in lethally irradiated mice allowed their differentiation into neuronal and astroglial cells [45]. Interestingly, the systemic injection of MSCs in non-irradiated but brain-lesioned mice had positive effects on injury repair, but very few MSC-derived cells expressed neural marker in such conditions [22,33,34,46,47]. Given those results, somatic stem cells raise thus hopes in cell replacement strategies based on an autograft approach. Recent *in vivo *studies [30,31] demonstrated that MSCs adopt neural fate by fusion with host neural cells. At least so far, there is no a clear and conclusive demonstration of a real differentiation of MSCs in neural cells. However, all those data obtained in vivo suggested that somatic stem cells seem to be promising for cellular therapy in neural diseases whatever the mechanism responsible for. It remains that a better knowledge of the molecular and cellular mechanism underlying the neural phenotypic plasticity of MSCs is required before considering those cells in human graft protocols.

As we mentioned above, environment is able to modify the cellular differentiation capacity. In this study, we addressed the question of a possible effect of MSCs on neural progenitor cell fate and we choose an *in vitro *approach. Co-culture experiments demonstrate that MSCs display multiple activities in the regulation of embryonic striatum-derived progenitors and stem cells. Their effect mainly depends of their age *in vitro*: npMSCs (more than 10 passages *in vitro *or 25 doubling cell populations) increase the astrocytes numbers while inhibiting neuronal and oligodendroglial numbers. Conversely, nnMSCs (with a maximum of 5 passages *in vitro*) only decrease the neuronal differentiation. Since similar results were obtained using MSC conditioned media, we concluded that these effects could be mediated by diffusible factor(s). We then analysed the effect of various conditioned media on the cell proliferation (using BrdU incorporation) as well as the cell death (using propidium iodide incorporation) in each neurosphere-derived cell types and at two different differentiation period (48 or 96 hours). We only observed a significant decrease of propidium iodide incorporation in GFAP-positive cell population at 48 hours of differentiation suggesting that soluble factor(s) select astroglial lineage by protecting those cells from cell death.

The activity which drives neurosphere-derived cells into astrocytes only occurs in npMSC conditioned medium while the neuronal differentiation-inhibiting activity is present both in np- and nnMSC conditioned media. We tried to identify the astrogliogenetic factor(s) by measuring the expression by nn- and npMSCs of cytokines known to promote an astroglial fate [36-41]. Our results show that only npMSCs express mature and biologically-active BMP4 while a biologically-inactive BMP4 precursor form is expressed and released by nnMSCs and neurosphere-derived cells in culture. Indeed, BMP4 is synthesized and released as an inactive precursor before being proteolytically activated by cleavage at the amino acid motif -Arg-Ser-Lys-Arg- [48]. Relatively little is known about the regulatory mechanisms controlling the susceptibility of individual TGF-β family members to proteolytic cleavage. However, recent studies suggest that members of the subtilisin-like proprotein convertase (SPC) family, SPC1 and SPC4, could enhance the cleavage of BMP4 precursor. The availability of biologically active BMPs may therefore be controlled by the released of their precursor followed by the action of the proprotein convertases [49,50].

The release by npMSCs of biologically-active form of BMP4 which promotes astroglial differentiation and inhibits oligodendroglial differentiation is consistent with previous studies demonstrating that BMPs play multiple roles in development [8]. Other studies have reported that the effects of BMPs are age- and tissue-dependent [51,52] and that BMPs promote astroglial differentiation and inhibit oligodendroglial differentiation when applied to cultures of cortical cells plated at E16 [23]. Likewise, brief treatment with BMPs induces astroglial fate in cultured neural precursors from embryonic mice [37] or in oligodendrocytes from newborn rats [51]. More recently, Rajan et al. [41] demonstrated that BMP4 induces astroglial differentiation of E14 and adult cortical neural stem cells from the subventricular zone when they are placed in high density culture. In this case, BMP4 has a true instructive role as NSCs cultures were clonal. Beside the instructive effect of BMP4 on glial lineages, some studies explained how BMP4 could act on astrocytic and oligodendrocytic precursors. A study realised on cerebellar primitive neurectodermal tumor cell line demonstrated that a high concentration of BMP2 and BMP4 attenuate apoptosis [53]. BMP-mediated inhibition of oligodendrogenesis is controlled through the repression of the former transcription factor olig2 known to be essential for the oligodendrocytic development [54,55]. All of these data suggest that BMP4 released by npMSCs selectively act on astrocytic and oligodendrocytic progenitors. In our experiments, we only demonstrate the anti-apoptotic effect of npMSCs-conditioned medium but we didn't test its possible instructive effect in clonal cultures. As BMP4, present in a biologically-active form in npMSCs conditioned medium, has been identified to be responsible of the increase of astrocytes numbers by the immunoblocking experiment and as it has been already demonstrated to be instructive [41], one could hypothesise that, in our system, the increase of astrocytes in response to npMSCs-derived BMP4 is a consequence of both a anti-apoptotic effect on GFAP-positive cells and also an instructive effect on NSCs.

## Conclusions

When considering the use of MSCs in cell replacement strategies for the treatment of various neurological diseases, it should be taken into account that those cells could also influence the development host neural precursors.

## Methods

### Preparation and culture of rat mesenchymal stem cells (MSCs)

Adult rat bone marrow was obtained from femoral and tibial bones by aspiration and was resuspended into 5 ml of DEM (Invitrogen, Merelbeke, Belgium) [56]. Between 100 and 200 × 10^6 ^marrow cells were plated on 175-cm^2 ^tissue culture flask in DEM/10% foetal bovine serum (Invitrogen). After 24 hours, the non-adherent cells were removed. When the rMSCs became confluent, they were resuspended with 0.25% Trypsin and 1 mM EDTA and then sub-cultured. Nestin expression by MSCs was induced as described in [32].

### Preparation and culture of mouse striatal neural progenitor and stem cells

Green C57BL/6 mice embryos (Jackson Immunoresearch Laboratory, Inc., West Grove, USA) or NMRI mice were used as a source of striatal neural progenitor and stem cells. Green mouse express GFP under control of the β-actin promoter [57]. The day of conception was determined by the presence of a vaginal plug (embryonic day 0). E16 striata were isolated and triturated in DEM/F12 (Invitrogen) with a sterile Pasteur pipette. The cell suspension was filtered with a 70 μm-mesh and viable cells were estimated by trypan blue exclusion. The cells were plated (1 × 10^6 ^cells/75-cm^2 ^tissue culture flask) in DEM/F12 supplemented with EGF (20 ng/ml, Sigma) and B27 (Invitrogen), a multi-component cell culture supplement devoid of any growth factor. When the size of neurospheres reached approximately 50 cells, they were dissociated into a single cell suspension by trituration and replated in fresh culture medium. Neurospheres with a maximum of 3 passages were used in this study.

### Co-culture of MSCs and neurospheres

MSCs and neurospheres were plated on polyornithine coated dishes for 5 days, in DEM/F12 containing only B27 supplement and were then processed for immunocytochemical analysis as described below.

### Culture of neurospheres in MSCs conditioned medium

MSCs were placed in DEM/F12 medium supplemented with B27 (Invitrogen), during 3 days. The conditioned media were then filtered with 0.22 μm-pore filter before being replaced on plated neurospheres during 5 days.

### Immunocytochemistry

The cultures were fixed with 4% (v/v) paraformaldehyde for 15 minutes at room temperature and washed 3 times in TBS buffer. They were then permeabilized in 1% Triton-X100 (v/v) for 15 minutes and washed 3 times in TBS buffer. Non-specific binding was blocked by a 1 hour treatment in TBST (TBS buffer with 0.1% Tween) containing defatted milk powder (30 mg/ml). The cells were then incubated for 1 hour at room temperature with either anti-glial fibrillary acidic protein (GFAP, Dako, mouse IgG, dilution 1:500), or Tuj1 (Molecular Probes, mouse IgG, dilution 1:1000), or O4 (Chemicon, mouse IgM, dilution 1:100) primary antibodies (diluted in blocking buffer). After 3 washes in TBS, cells were then incubated in FITC- or Cy5-conjugated anti-mouse IgG or IgM (Jackson Immunoresearch, 1:500) for 1 hour at room temperature and in the dark. The nuclei were stained with ethidium homodimer (0.2 μM, Sigma). The preparations were then mounted in Fluoprep™ (Biomerieux; France) and observed using a Bio-Rad MRC1024 laser scanning confocal microscope. The fraction of positive cells was determined by counting 10 non-overlapping microscopic fields (±50 cells/field, ±3500 cells/experiments) for each coverslip in at least three separate experiments (n then corresponds to the number of coverslips).

### BrdU and propidium iodide incorporation

After 24 hours or 3 days of culture, BrdU (20 μM, Sigma) which is a S-phase marker, or propidium iodide (400 mg/ml, Sigma) was added to the differentiating neurosphere cultures for 18 hours before fixation and staining. Tuj1, O4 and GFAP immuno-labellings were performed as described above. For BrdU labelling, coverslips were then post-fixed for 10 minutes in 4% (v:v) paraformaldehyde, incubated in HCl 1 N for 20 minutes at 37°C, washed with sodium perborate solution (50 mM, pH 8.5) and finally incubated with an anti-BrdU antibody for 1 hour at room temperature (Oxford, rat IgG, dilution 1:200) and Cy-5-conjugated anti-rat antibody, 1 hour at room temperature. The preparations were analysed as describe above.

### RNA extraction and quantitative RT-PCR analysis

Total RNA was prepared using the RNeasy total RNA purification kit (Qiagen, Westburg). For cDNA synthesis, random hexamer primers (Invitrogen) were used to prime reverse transcriptase reactions. The cDNA synthesis was carried out using Moloney-murine leukemia virus (M-MLV) Superscript II Reverse transcriptase (Invitrogen) following the manufacturer's instructions. Quantitative PCR was carried out using standard protocols with Quantitec SYBR Green PCR Kit (Qiagen). The PCR mix contained SYBR Greeen Mix, 0.5 μM primers (Table 1), 1 ng DNA template and nuclease free water to final volume of 25 μl. PCR were performed on RotorGene RG-3000 (Corbett Research) and analyzed with Rotorgene Software (Corbett Research). The percentage of gene expression by npMSCs was normalized in function of GAPDH gene expression and compared to the gene expression by nestin-negative MSCs that was considered as 100%. Each gene analysis was realised on three different samples with two run/sample (we then provided 6 PCR analyses by target gene)

### Western Blot

After 72 hours of culture, the conditioned medium (1.5 ml) from cultures of neural progenitor cells, npMSCs and nnMSCs were incubated with heparin sepharose CL-6B (Amersham Biosciences, Belgium) at 4°C for 24 hours. After centrifugation (700 × g), the supernatant was removed and bound proteins were eluted in loading buffer (glycerol 10% v/v; Tris 0.05 M pH 6.8; SDS 2%, bromophenol blue and 2.5% v/v – mercaptoethanol) by heating at 70°C for 10 minutes. The protein concentrations of various samples were quantified using the "RC DC Protein Assay" (Bio-Rad, Belgium) and equal protein quantities were loaded in each lane in a 15% sodium dodecyl sulfate polyacrylamide gel and electrophoresed. Then the proteins were transferred to PVDF membrane (Amersham Biosciences, Belgium) using a Trans-Blot Semi-Dry Transfer apparatus (Bio-Rad). The membranes were saturated with 3% gelatin (BioRad) during 1 hour at 37°C, then incubated for 1 hour with a monoclonal antibody against BMP4 (R&D, goat IgG, 0.1 μg/ml) or actin, as control for protein loading (Sigma, mouse IgG 1:5 000) at room temperature and then washed several times with PBS-0.1% Tween. The membrane was then incubated in Cy5-conjugated anti-goat IgG or anti-mouse IgG (Jackson Immunoresearch, 1:2500) for 1 hour at room temperature, in the dark. After several washes in PBS, the membranes were scanned using a Typhoon 9200 Scanner (Amersham Biosciences) and subsequent analyses were performed with ImageQuant Software (Amersham Biosciences).

### BMP4 neutralization

Neurospheres were plated on polyornithine-coated dishes and incubated during 5 days in DEM/F12 medium supplemented with B27 (Invitrogen) and previously conditioned by npMSCs during 3 days or not. Anti-BMP4 antibody (R&D, 2 μg/ml) was added on day 1 of this incubation.

## Authors' contributions

SW performed most of the work and wrote a first draft of the manuscript; FB performed quantitative RT-PCR; GH, GM and PL were involved in the writing of the manuscript; BR conceived the study and participated in its design and coordination. All authors read and approved the final manuscript.

## Figures and Tables

**Table 1:** Primer list used for quantitative RT-PCR

Primers	Sequence	Tm
BMP2 Forward	CCAAGGTTAGTGACTCAGAACAC	52
BMP2 Reverse	TCATCTTGGTGCAAAGACCTGC	52
BMP4 Forward	ACTGCCCCACCTTCTCTGAG	50
BMP4 Reverse	TTCTCCAGATGTTCTTCGTG	50
LIF Forward	GCAACCTCATGAACCAGATC	52
LIF Reverse	CAACTTGGTCTTCTCTGTCC	52
CNTF Forward	ATTCGTTCAGACCTGACTGC	50
CNTF Reverse	CCTGATGGAAGTCACCTTCA	50
GAPDH forward	GACCCCTTCATTGACCTCAACTACATG	48–55
GAPDH Reverse	GCCTTCTCCATGGTGGTGAACAC	48–55
